# Inferring responses to climate warming from latitudinal pattern of clonal hybridization

**DOI:** 10.1002/ece3.5896

**Published:** 2019-12-04

**Authors:** Katherine Monette, Christelle Leung, Joelle Lafond, Julian Wittische, Bernard Angers

**Affiliations:** ^1^ Department of Biological Sciences Université de Montréal Montreal QC Canada

**Keywords:** *Chrosomus eos–neogaeus* complex, clonal hybrid, global warming, hybridization, latitudinal distribution, premating barriers

## Abstract

Climate warming may affect reproductive isolation between sympatric sister species by modifying reproductive phenology or mate choice. This is expected to result in a latitudinal progression of hybridization in response to the shifting of environmental conditions. The fish species northern redbelly dace (*Chrosomus eos*) and finescale dace (*C. neogaeus*) display a wide sympatric distribution in North America. The asexual reproduction of their hybrids allows determining where and when hybridization occurred. The aim of this study was twofold: first, to assess whether temperature affected reproductive isolation, and second, whether the effects of climate warming resulted in a latitudinal progression of hybridization. We performed a 500 km latitudinal survey (51 sites) in southeastern Quebec (Canada) and determined the distribution of clonal hybrid lineages. Results revealed a total of 78 hybrid lineages, including 70 which originated locally. We detected a significant difference between the southern and northern range of the survey in terms of the proportion of sites harboring local hybrids (20/23 vs. 8/28 sites, respectively) and hybrid diversity (57 vs. 13 lineages, respectively). This confirmed that there was more frequent interspecific mating in the warmest sites. In the southern range, diversity of lineages and simulations suggest that hybridization first took place (>7,000 years) in sites characterized by a longer growing season, followed by northerly adjacent sites (ca. 3,500–5,000 years). Moreover, evidence of hybridization occurring in present‐day time was detected. This suggests that the current warming episode is going beyond the limits of the previous warmest period of the Holocene.

## Graphical abstract

The asexual reproduction of *Chrosomus eos–neogaeus* hybrids allows determining where and when hybridization occurred, a useful characteristic to assess whether climate warming affects reproductive isolation. A 500 km latitudinal survey in southeastern Quebec (Canada) revealed a significant difference in terms of the proportion of sites harboring local hybrids and hybrid diversity, between the southern and northern halves of the survey. This more frequent interspecific mating in the warmest sites and evidence of hybridization occurring in present‐day time suggest that the current warming episode is going beyond the limits of the previous warmest period of the Holocene.

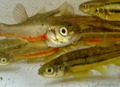

## 1 INTRODUCTION

Global warming refers to the general increase in temperature across the planet since the late 19th century. The modifications of environmental conditions brought by the rise of the temperature have major impacts on premating barriers, making hybridization a serious consequence of global warming (Chunco, 2014; Hoffmann & Sgro, 2011; Parmesan & Yohe, 2003; Scheffers et al., 2016). Firstly, shift in the geographic distribution of species is one response to the current climate warming (Chen, Hill, Ohlemüller, Roy, & Thomas, 2011; Kelly & Goulden, 2008) and also occurred during postglacial expansion (e.g., Dyke, 2005). Secondary contact resulting of a differential shift in the range of closely related species may, however, lead to the formation of a hybrid zone and have been reported for several examples (e.g., Ryan et al., 2018). Secondly, climate warming can also affect reproductive phenology or mate choice and weaken reproductive isolation between sympatric nascent species (reviewed in Chunco, 2014). The reproductive phenology of organisms is strongly influenced by temperature, and warmer conditions often result in earlier time of mating (Benard, 2015; Carey, 2009; Walther et al., 2002; Wegge & Rolstad, 2017). Differential shift in the timing of reproduction may lead to an overlapping of reproductive periods between species and to hybridization (Chunco, 2014; Vallejo‐Marín & Hiscock, 2016). Warming can affect mate choice when habitats become unfavorable to one species. This may increase the probability of hybridization due to asymmetric availability of mates. Indeed, according to the Hubbs principle (Hubbs, 1955), a species decreasing in abundance is expected to breed with individuals of another more abundant species as conspecific individuals become scarce (Avise & Saunders, 1984; Grant & Grant, 1997; Jansson, Thulin, & Pehrson, 2007; Randler, 2002; Wirtz, 1999).

The effect of warming is not uniform across the latitudinal range of species because temperature varies with latitude (and altitude). If temperature must reach a threshold to trigger a given change, warmer regions will be first affected, and with time (and continuous warming), the geographic limit reaching this threshold will move latitudinally. The modification of premating barriers between sympatric related species is therefore expected to result in a wave of hybridization through the sympatric area in response to climate warming.

Moreover, the current climatic warming is not an isolated event. The transition from Pleistocene to Holocene ca. 12,000 years BP was marked by a strong increase in temperature, while the following Kyears were punctuated by cooling and warming episodes (Marcott, Shakun, Clark, & Mix, 2013). For instance, the Holocene Climate Optimum was a period characterized by the warmest temperatures since the end of the Pleistocene, with conditions warmer than now (but see Porter et al., 2019). The time of Holocene Climate Optimum varied widely across the planet (Kaufman et al., 2016; Marcott et al., 2013). For instance, this peak warming occurred ca. 6,000–3,500 years BP in northeastern North America (Comtois, 1982; Larochelle, Lavoie, Grondin, & Couillard, 2018).

**Figure 1:**
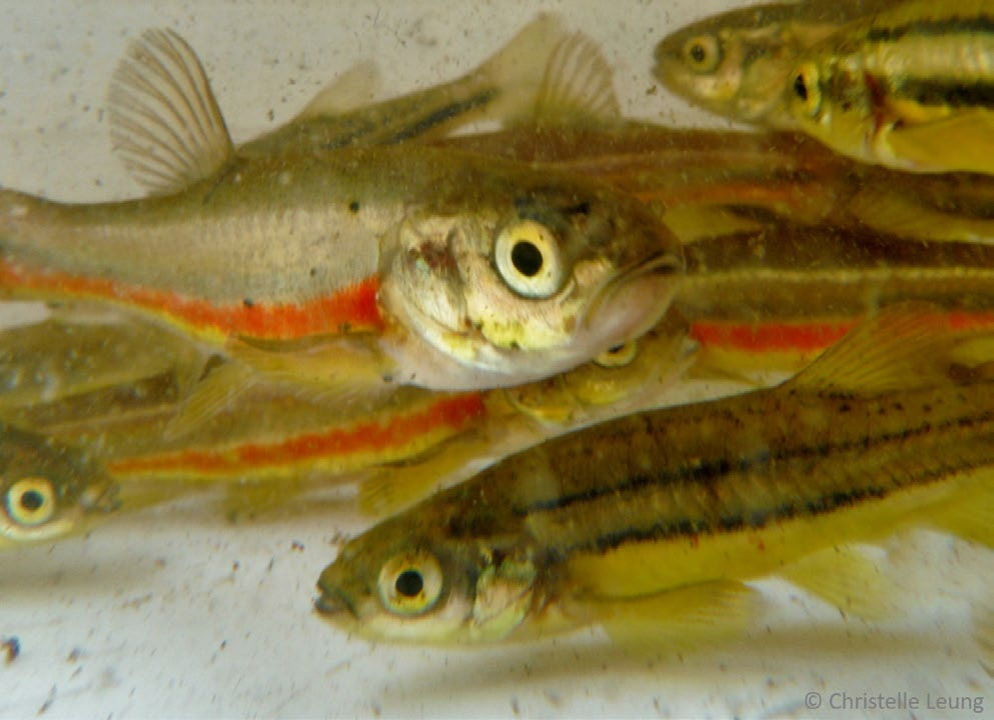
The northern redbelly dace (*Chrosomus eos*) and the finescale dace (*C. neogaeus*). Clonal hybrids *C. eos–neogaeus* (absent in the picture) result from hybridization events between those species. The northern redbelly dace has two black stripes alongside (right side of the picture), while the finescale dace exhibit a thick orange stripe (left side of the picture)

To determine the extent of historical warming events and assess the magnitude of the current episode on hybridization, the clonal hybrids represent a useful biological indicator through which to infer the effects of climate warming since they keep track of hybridization events. The northern redbelly dace (*Chrosomus eos*; Figure 1) and the finescale dace (*C. neogaeus*; Figure 1) are fish species with a wide overlapping distribution in the north of North America, mostly located in regions previously covered by the last Pleistocene ice sheet (Scott & Crossman, 1973). Distinct spawning times are expected to provide a premating barrier between those species (Das & Nelson, 1990). However, this temporal barrier is not completely impervious and *C. eos–neogaeus* hybrids are also widely distributed (Angers & Schlosser, 2007). A particular characteristic of these hybrids is that they are only females and reproduce clonally, as observed in several other unisexual hybrid vertebrates (Neaves & Baumann, 2011). The absence of meiosis in the hybrid genome prevents backcrosses and genetic exchanges between *C. eos* and *C. neogaeus* species (Lafond, Hénault, Leung, & Angers, 2018). Therefore, once hybrid individuals are produced, their genotypes can persist through time via clonal lineages.

Surprisingly, most of those hybrids do not originate from local hybridization, but rather predated the end of the Pleistocene. Indeed, previous studies have reported the presence of a single or a few widespread lineages in most of the regions surveyed (Angers & Schlosser, 2007; Goddard, Dawley, & Dowling, 1989). However, both species can still interbreed and local hybridization events have been genetically confirmed in the southeastern region of Quebec, Canada (Vergilino, Leung, & Angers, 2016). Compared to Pleistocene migrants, local hybridization resulted in several lineages specific to a given site (private lineages). These assumptions parallel those inferred from mitochondrial DNA in phylogeographic studies of regions formerly covered by glaciers (e.g., Bernatchez & Wilson, 1998). mtDNA is asexually transmitted as are the clonal hybrid lineages. On the one hand, mtDNA haplotypes already present in glacial refuges displayed a large geographic distribution. They took advantage from the proglacial lakes and temporary bridges among hydrographic networks to spread throughout different watersheds (Curry, 2007; Mandrak & Crossman, 1992; Parent & Occhietti, 1999). On the other hand, mtDNA variants that appeared more recently are generally restricted to the contemporary drainage system as interconnections are no longer present.

Since local hybridization events appear to be rare in most sperm‐dependent unisexual hybrids, this raises questions about the causes of such high rates of spontaneous hybridization in that region. The aim of this study was twofold: first, to assess whether temperature (or other correlated environmental conditions) affected reproductive isolation between *C. eos* and *C. neogaeus* (spatial pattern), and second, whether effects of climate warming (current and/or historical) resulted in a latitudinal progression of hybridization through time (temporal pattern). To address these objectives, we performed a 500 km latitudinal survey in the southeastern Quebec region to investigate the large‐scale geographic variation in distributions of the *C. eos–neogaeus* hybrids, as a consequence of local hybridization between *C. eos* and *C. neogaeus*.

Clonal hybrids are not only useful to assess local hybridization that occurred thousands of years ago, but they could also be used to infer time since hybridization. This would be possible if hybridization is not a continuous process, so in the absence of new hybrids, lineage diversity decreases with time. Complete extirpation of one parental species stops hybridization. Such a scenario is one conclusion of a species decline associated with hybridization according to the Hubbs principle (Hubbs, 1955). However, the limited occurrence of local hybridization when both species co‐occur suggests that other factors may prevent hybridization. *Chrosomus neogaeus* first reproduces in early spring, while *C. eos* reproduces later (Das & Nelson, 1990). The hybrid lineages analyzed so far were always the result of reproduction between late *C. neogaeus* females and early *C. eos* males, since all hybrids detected so far harbor the maternally inherited mitochondrial DNA of *C. neogaeus* (Angers & Schlosser, 2007; Binet & Angers, 2005; Goddard et al., 1989; Vergilino et al., 2016). Hybrids are known to display an intermediate reproduction period between that of *C. neogaeus* and *C. eos*. This reproduction period must overlap with that of one of the parental species because the reproduction of the hybrids requires the sperm of a parental species to activate the development of their eggs (gynogenesis). In addition, the asexual reproduction of all‐female clonal hybrids is expected to result in a rapid demographic expansion of hybrid population (Leung & Angers, 2018). The high abundance of sperm‐dependent female hybrids compared to the low number of remaining late *C. neogaeus* females may prevent or strongly reduce the probability of further hybridization events.

If hybridization is a punctual event rather than a continuous process, the number of hybrid lineages is expected to decrease with time in the absence of new hybridization events. A single interspecific mating generates a high diversity of lineages since each zygote producing a female can evolve as a distinct hybrid lineage (no androgenesis was reported in this species complex). However, through generations, random processes related to lineage sorting, as well as natural selection, progressively reduce the number of lineages. Therefore, the diversity of lineages at a given site depends on the time elapsed since the hybridization events (in addition to population size and environmental conditions). Relative time since local hybridization events can be correlated to lineage diversity.

In this contextual framework, we can formulate specific predictions for each objective. First, we expect a higher occurrence of hybridization in southern sites than in northern sites due to the higher temperature at lower latitude (closer to the threshold expected to weaken premating barriers). In northern sites, where premating barriers are not yet disturbed, the presence of locally produced hybrids is expected to be infrequent, even in the presence of sympatric parental species. Second, if climate warming (previous episodes during Holocene as well as the current episode) modifies premating barriers, hybridization is expected to progress northward through time. We therefore predict that the hybrid diversity resulting from local hybridization will vary according to a latitudinal gradient. Since the number of lineages decreases with time since hybridization, diversity is expected to increase going northward, as hybridization events are more and more recent.

## 2 METHODS

### 2.1 Sampling sites

**Figure 2:**
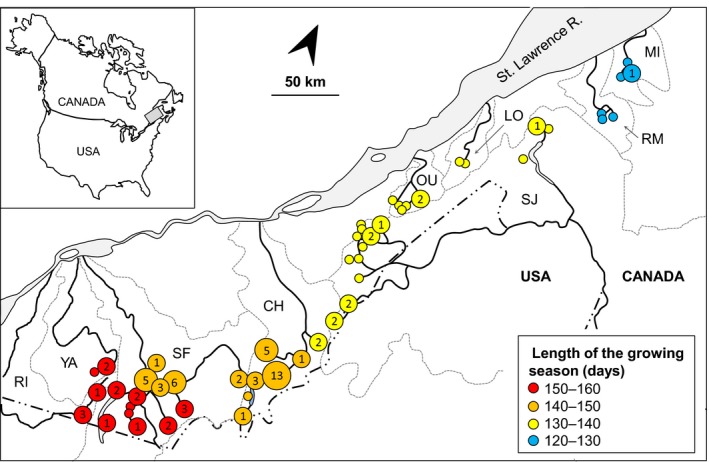
Map of southeastern Quebec (Canada) with the geographic location of sampling sites. Circles indicate sites in which *Chrosomus eos*, *C. neogaeus*, and/or hybrid *C. eos–neogaeus* individuals were sampled. The color of the circles refers to the length of the growing season (days) and the numbers and the size to the absolute abundance of private hybrid lineages at that site (absence of number indicates a site without private hybrid lineage). Dotted lines delimit the drainage basin of the Richelieu (RI), Yamaska (YA), Saint Francois (SF), Chaudière (CH), Saint John (SJ), Ouelle (OU), Du Loup (LO), Rimouski (RM), and Mitis (MI) rivers. All these systems belong to the St. Lawrence River drainage except for SJ, which drains into the Atlantic Ocean. Insert indicates the geographic position of the study site in North America

We analyzed a total of 51 sites along a 500‐km‐long transect, from 45°02′ to 48°29′ north (Figure 2, Table 1). Of these, 19 sites are representative of the southernmost hydrographic networks of the southeastern Quebec: Richelieu R. (RI), Yamaska R. (YA), and Saint Francois R. (SF) sampled in previous studies (Leung, Breton, & Angers, 2016; Vergilino et al., 2016). Nine of these 19 sites were a pool of geographically close sites located in the same network and harboring the same hybrid assemblage, to avoid over‐representation of these assemblages representing the same local hybridization events.

**Table 1:** Characteristics of the sampled sites

Site	Latitude	Longitude (W)	Biotypes			# lineages	Shared lineages	LGS (days)	Sampling
	(N)		E	H	N				
RI‐2‐4	45°03′01ʺ	72°19′03ʺ	31	40	0	3		150–160	1
SF‐3, 18	45°03′05ʺ	72°11′39ʺ	0	5	0	1		150–160	1
SF‐11	45°04′23ʺ	71°52′37ʺ	0	2	0	1		150–160	1
SF‐12	45°07′48ʺ	71°40′21ʺ	28	51	12	2		150–160	1
SF‐13, 14	45°11′04ʺ	71°33′13ʺ	14	114	0	3 (1)	A	150–160	1
SF‐9‐10	45°12′23ʺ	71°56′33ʺ	30	0	0			150–160	1
SF‐6	45°13′20ʺ	71°55′01ʺ	9	0	0			150–160	1
SF‐4,5,7,8	45°14′01ʺ	71°54′28ʺ	12	54	0	2		150–160	1
RI‐1	45°15′03ʺ	72°18′20ʺ	0	4	0	1		150–160	1
SF‐2	45°21′17ʺ	72°13′05ʺ	14	7	0	2		150–160	1
CH‐1	45°22′48ʺ	70°50′02ʺ	0	1	3	1		140–150	1
YA‐1	45°23′23ʺ	72°27′01ʺ	10	24	0	2 (1)	B	150–160	1
YA‐2	45°24′25ʺ	72°25′18ʺ	24	0	0			150–160	1
SF‐15	45°24′53ʺ	71°46′53ʺ	0	6	0	3		140–150	1
SF‐20, 21	45°25′50ʺ	71°40′19ʺ	2	24	0	6		140–150	1
SF‐1	45°27′00ʺ	71°49′32ʺ	16	14	0	5 (1)	A	140–150	1
CH‐2	45°29′03ʺ	71°04′48ʺ	80	0	0			140–150	1
SF‐19, 22	45°32′15ʺ	71°49′40ʺ	0	12	0	1 (1)	B	140–150	1
CH‐3	45°41′10ʺ	70°55′01ʺ	1	47	0	3 (2)	C, D	140–150	2
SF‐16,17	45°42′10ʺ	71°09′59ʺ	0	6	0	2		140–150	1
CH‐4	45°46′59ʺ	70°51′13ʺ	13	24	4	13 (2)	D, E	140–150	2
CH‐5	45°50′45ʺ	70°53′19ʺ	19	16	29	5 (1)	E	140–150	2
CH‐6	45°56′02ʺ	70°47′37ʺ	3	4	0	1		140–150	2
CH‐7	46°06′29ʺ	70°24′50ʺ	7	21	0	2 (1)	C	130–140	2
SJ‐1	46°10′52ʺ	70°18′19ʺ	10	15	11	2 (1)	F	130–140	2
SJ‐2	46°11′22ʺ	70°18′40ʺ	21	16	7	2 (1)	F	130–140	2
SJ‐3	46°20′40ʺ	70°14′48ʺ	8	2	0	0 (1)	G	130–140	2
SJ‐4	46°32′42ʺ	70°14′38ʺ	5	3	2	0 (2)	F, G	130–140	2
SJ‐5	46°39′05ʺ	70°14′35ʺ	0	4	23	0 (1)	F	130–140	2
SJ‐6	46°40′19ʺ	70°10′16ʺ	4	0	0			130–140	2
SJ‐7	46°48′54ʺ	70°13′45ʺ	21	12	0	2 (2)	F, G	130–140	2
SJ‐8	46°51′27ʺ	70°10′12ʺ	1	11	10	1 (2)	F, H	130–140	2
SJ‐9	46°51′45ʺ	70°10′37ʺ	17	2	3	0 (1)	G	130–140	2
SJ‐10	46°54′33ʺ	70°14′23ʺ	3	8	0	0 (3)	F, G, H	130–140	2
SJ‐11	46°55′50ʺ	70°04′22ʺ	4	0	0			130–140	2
OU‐1	47°05′23ʺ	69°51′50ʺ	17	4	3	0 (2)	F, H	130–140	2
OU‐2	47°06′31ʺ	69°53′52ʺ	15	2	9	0 (1)	H	130–140	2
OU‐3	47°06′32ʺ	70°00′30ʺ	9	0	7			130–140	2
OU‐4	47°07′33ʺ	69°50′40ʺ	0	2	0	0 (1)	F	130–140	2
OU‐5	47°09′12ʺ	69°48′26ʺ	25	22	1	2		130–140	2
LO‐1	47°30′44ʺ	69°32′24ʺ	12	0	0			130–140	2
LO‐2	47°32′15ʺ	69°35′07ʺ	8	0	4			130–140	2
SJ‐12	47°42′05ʺ	68°59′36ʺ	0	6	0	1		130–140	2
SJ‐13	47°55′03ʺ	68°57′16ʺ	0	0	1			130–140	2
SJ‐14	47°55′41ʺ	68°50′53ʺ	1	0	0			130–140	2
RM‐1	48°05′02ʺ	68°32′47ʺ	2	0	0			120–130	2
RM‐2	48°06′43ʺ	68°31′50ʺ	2	0	0			120–130	2
RM‐3	48°08′05ʺ	68°25′04ʺ	12	0	6			120–130	2
MI‐2	48°18′15ʺ	68°14′32ʺ	2	0	0			120–130	2
MI‐1	48°19′33ʺ	68°18′54ʺ	3	0	1			120–130	2
MI‐3	48°29′38ʺ	68°14′27ʺ	16	1	0	1		120–130	2

Note

List of sampled sites with their geographic coordinates and sample size of each biotype (E, N, and H refer to *Chrosomus eos*, *C. neogaeus*, and the hybrids, respectively). The number of private hybrid lineages (and shared lineages) of each site is indicated. Lineages shared among sites are indicated by the same letter. LGS refers to the length of the growing season in days according to Agriculture and Agri‐Food Canada (2014). Sampling refers to sites sampled 1—Vergilino et al. (2016) and 2—this study. Each site is designated by two letters referring to its hydrographic networks. Sites with more than one number (e.g., RI 2‐4) indicated sites pooled together.

In addition, individuals from 32 new sites were sampled in six additional hydrographic networks: Chaudière R. (CH) northerly adjacent to SF, then moving northward Saint John R. (SJ), Ouelle R. (OU), Du Loup R. (LO), Rimouski R. (RM), and Mitis R. (MI).

This research was performed under institutional animal care guidelines (permits #13‐084 and 18‐019 delivered by the Université de Montréal) and conforms to the mandatory guidelines of the Canadian Council on Animal Care. Sampling permits were provided by the Quebec Ministry of Natural Resources and Wildlife (MRNF).

This transect encompassed a broad latitudinal range and temperature gradient. For instance, there is a difference of 3.0°C in the mean annual temperature between the southernmost and the northernmost sites. The length of the growing season (LGS) was used to represent the latitudinal variation of temperature and other associated environmental conditions for which it can be used as a proxy. Indeed, the LGS is limited by different factors, such as air temperature, frost days, rainfall, and daylight hours; it provides an integrative index of climate differences among regions (Linderholm, 2006) and is often correlated to phenotypic change in animals (Garel, Solberg, SÆther, Herfindal, & Høgda, 2006; Peñuelas, Filella, & Comas, 2002; Zani, 2008). Increasing temperature is expected to lengthen the growing season of temperature‐limited organisms such as ectotherms. The LGS was measured in days, starting from 10 days after average daily temperature is above 5°C and ending when the minimum daily temperature is 0°C or October 31st is reached—whichever comes first and has been estimated from records, using a 29‐year (from 1971 to 2000) reference period of measure (Agriculture & Agri‐Food Canada, 2014). We used the map of Agriculture and Agri‐Food Canada (2014) where LGS categories are incremented by 10 days, to assign our sampling sites to one or the other categories (Figure 2). A difference of 40 days in the LGS was observed between the southernmost and the northernmost sites. Although the climatic conditions were likely different than those during the Holocene, these categories were used to emphasize the latitudinal differences along our 500 km survey.

### 2.2 Diversity of hybrid lineages

External morphological characteristics were used to visually identify sampled individuals as *C. eos*,* C. neogaeus*, or hybrids (New, 1962). DNA was extracted from a small piece of the upper lobe of the caudal fin, and we confirmed the visual identification using the genetic markers specific to nuclear and mitochondrial genomes developed by Binet and Angers (2005).

For all individuals identified as hybrids, a multilocus genotype was determined according to the size variation of microsatellite loci Seat412, Ca‐12, Pho‐60, Pho‐1, Pho‐2, and Rhca‐20 (Angers & Schlosser, 2007; Binet & Angers, 2005; Skalski & Grose, 2006). Loci Seat412, Ca‐12, and Rhca‐20 amplify both *C. eos* and *C. neogaeus* genomes, while Pho‐60, Pho‐1, and Pho‐2 are specific to the *C. eos* genome. Therefore, hybrid genotypes were characterized by a total of nine specific haplomes (six haplomes for *C. eos* and three haplomes for *C. neogaeus*).

A hybrid lineage was defined by individuals originating from the fertilization of one *C. neogaeus* egg by a *C. eos* sperm. As the clonal reproduction of hybrids leads to the absence of recombination and segregation, individuals of a given lineage are expected to display the same multilocus genotype (Angers & Schlosser, 2007). However, due to the high mutation rate of microsatellite loci (Estoup & Angers, 1998), variants could be detected. They generally differed by a single‐step mutation at one or a few loci. A lineage was thereby characterized by a consensus genotype defined by the allele of the invariant loci and the most abundant allele for the variable loci (Angers & Schlosser, 2007).

In the *Chrosomus eos–neogaeus* complex, a high proportion of triploid hybrids are produced by the incorporation of the sperm genome into the diploid hybrids’ eggs (Goddard et al., 1989; Leung & Angers, 2018). Since triploid hybrids display both the consensus multilocus genotype of a given hybrid lineage and the spermatozoid haplome, it was possible to identify the hybrid lineage from which they derived. However, when no match could be made between a triploid genotype and any of the consensus lineages, this triploid genotype was considered as an additional lineage for which it was not possible to determine the hybrid genotype.

### 2.3 Local hybridization versus pleistocene migrants

Once lineages were identified, the next step was to determine whether hybrids were migrants from Pleistocene populations or descendants of local hybridization. The occurrence of the same lineage through different hydrographic networks was considered as the signature of hybrid lineage from the Pleistocene (Angers & Schlosser, 2007; Vergilino et al., 2016). In contrast, hybrid lineages produced locally occurred in isolated hydrographic networks similar to those in the current landscape and were expected to display a narrow geographic distribution limited to distinct subdrainage basins (Vergilino et al., 2016). Lineages specific to a given site (private lineages) were therefore likely the result of local hybridization events.

### 2.4 Geographic distribution of species and hybrids

The evenness of species distribution (in terms of the proportion of sites occupied) across LGS categories was tested by Fisher's exact test, while the abundance of species was compared by a chi‐square test.

### 2.5 Spatial pattern of hybridization

To assess the effect of temperature on reproductive isolation of *C. eos* and *C. neogaeus*, the difference in the number of sites harboring private hybrid lineages (as indication of local hybridization) and diversity of private lineages were tested according to the LGS using Fisher's exact test and nonparametric Mann–Whitney *U* tests, respectively. The diversity of private lineages across a latitudinal gradient was also represented using a LOESS/LOWESS (Locally Weighted Scatter‐plot Smoother) regression (Cleveland, 1979; Cleveland, Grosse, & Shyu, 1991). The smoothing parameter was selected automatically using a generalized cross‐validation criterion (Golub, Heath, & Wahba, 1979), as implemented for fANCOVA package version 0.5‐1 (Wang & Wang, 2010) in the statistical programming environment R 3.4.0.

### 2.6 Temporal pattern of hybridization

To assess whether hybridization occurred first in the southernmost sites and then progressed northwardly, we compared the diversity of private lineages between sites from LGS of 150–160 days to those from LGS of 140–150 days using a chi‐square goodness‐of‐fit test.

Lineage diversity (in terms of the number of lineages) of one site (CH‐4) has been underestimated by the sampling due to the presence of several lineages with low frequency. To assess the effective number of lineages in this population, we estimated the number of lineages from 100 random samplings (*N* = 24) from populations generated by EASYPOP 1.7 (Balloux, 2001). Populations of 1,000 haploid individuals were produced, where variability of the initial population was set to maximum (lineages were randomly assigned, but keeping one lineage at a frequency of 0.33 as observed in the sampling of this site) using 15 (the number of lineages detected), 30, 40, or 50 lineages.

Finally, to illustrate the decrease in hybrid diversity over time, we simulated the random processes of lineage sorting using EASYPOP 1.7 (Balloux, 2001). We used 10,000 clonal individuals distributed in 20 populations of 500 individuals connected by migration (m = 0.01), representative of the natural populations living in small brooks. One hundred simulations were performed, starting with a maximum of diversity (100 lineages randomly assigned) and lasting 10,000 generations.

## 3 RESULTS

### 3.1 Geographic distribution of species and hybrids

**Figure 3:**
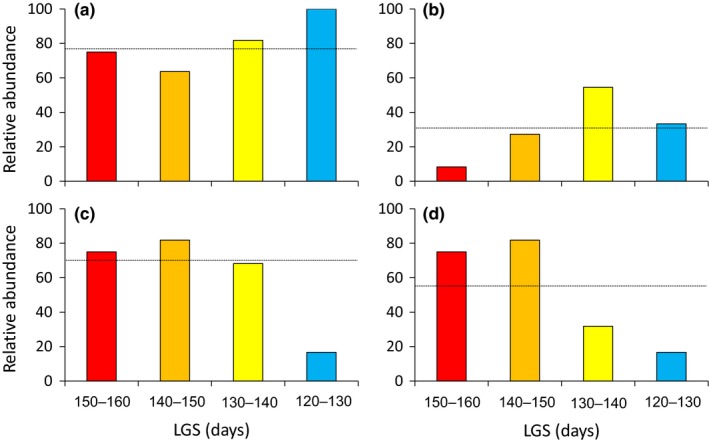
Geographic distribution of species and hybrids across length of the growing season (LGS) categories. The relative abundance of species and hybrids is expressed as the proportion of sites occupied by a biotype over all sites sampled in a given LGS category, for (a) the redbelly dace *C. eos*, (b) the finescale dace *C. neogaeus*, (c) all hybrid lineages, and (d) only private hybrid lineages. The horizontal bar indicates the mean overall sites

Both *C. eos* and *C. neogaeus*, as well as hybrids, were found throughout all hydrographic networks (Figure 3, Table 1). The sexual species *C. eos* was detected in most of the sites (40 out of 51 sites; 78.4%). This species displayed an even distribution; the proportion of sites occupied by *C. eos* did not differ among LGS categories (Fisher's exact test *p* = .3745).

The other sexual species *C. neogaeus* was only detected in 18 sites (35.3%) and was less abundant than *C. eos* (χ^2^ = 17.63; *df* = 1; *p* = .00003). The distribution of this species is uneven (Fisher's exact test *p* = .0429), being less abundant in the LGS of 150–160 days (Figure 3). For instance, this species occupied a single site in the southernmost networks (YA, RI an SF; Table 1).

The hybrids were detected in 36 sites (70.6%) and were as abundant as *C. eos* (χ^2^ = 0.46; *df* = 1; *p* = .4955). However, their distribution across LGS categories is also uneven (Fisher's exact test *p* = .0124). At the opposite of *C. neogaeus*, hybrids were less abundant in the northern sites (LGS of 120–130 days; Figure 3).

### 3.2 Local hybridization versus pleistocene migrants

The genetic survey of hybrid individuals revealed a total of 78 distinct lineages (Table 1); 41 lineages were detected in the new sites surveyed in this study from CH, SJ, OU, LO, RM, and MI networks, in addition to the 37 lineages previously detected in the YA, RI, and SF networks (Vergilino et al., 2016). Although the sampling size differs among sites, there is no correlation between the number of hybrid individuals sampled and the number of lineages detected (*R* ^2^ = 0.08; *p* = .1).

Hybrids could be discriminated into two categories according to their geographic distribution. On the one hand, eight lineages displayed a large geographic distribution, being shared between at least two distant sites (lineages designated from A to H, Table 1). At the most extreme, the lineages F, G, and H displayed a broad distribution (up to 100 km) and were found in four, five, and nine sites, respectively. Furthermore, two of these lineages (F and H) were present in distinct hydrographic networks (SJ and OU). The large geographic distribution of these lineages suggests that they are migrants and originated from hybridization events during the Pleistocene. On the other hand, the remaining 70 lineages (89.7%) were sampled in a single site or between geographically close sites displaying the very same hybrid assemblage. These hybrids were considered as private lineages and likely resulted from local hybridization.

### 3.3 Spatial pattern of hybridization

Private hybrid lineages were present in 28 sites (54.9%). Their distribution across LGS categories is also uneven (Mann–Whitney *U* tests *p* = .0003) due to the low proportion of sites with private lineages in both 120–130 and 130–140 days LGS categories (Figure 3). Indeed, we sampled less private hybrid lineages in the northern sites (8/28 sites from SJ, OU, LO, RM, and MI networks) compared to the southern sites (20/23 sites from the YA, RI, SF, and CH networks; Figure 2).

**Figure 4:**
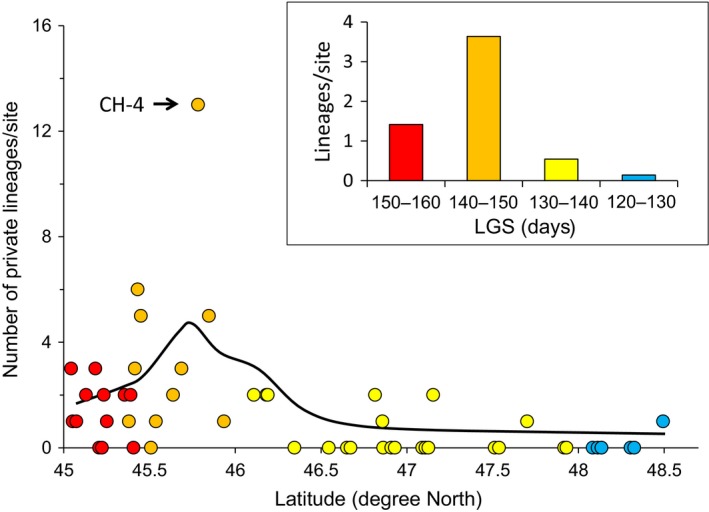
Relationship between hybrid diversity and latitude. Scatter plot and local regression curve between the number of private lineages and latitude. The color of the dots refers to the length of the growing season (LGS) (days). Insert indicates the diversity (mean number of private lineages per site) for each LGS category

We also detected a significant difference in term of hybrid lineage diversity across LGS categories (Figure 4; χ^2^ = 19.33 *df* = 3 *p* = .0002). A total of 57 private hybrid lineages (81.4%) were detected in the southern half of the survey (LGS of 140–150 and 150–160 days), contrasting with the northern half (LGS of 120–130 and 130–140 days) with only 13 hybrid lineages (Figures 2 and 4). This clear break in the diversity of hybrids indicates that there is more frequent interspecific mating occurring locally in southern sites characterized by a longer LGS than in the northern sites.

### 3.4 Temporal pattern of hybridization

We assessed the temporal change, more specifically whether hybridization first occurred in the southernmost sites (LGS of 150–160 days) before progressing northwardly (LGS of 140–150 days). Indeed, a higher diversity was observed in sites from LGS of 140–150 days (40 lineages, 3.63 lineages/site; 11 sites) than in southernmost sites from LGS of 150–160 days (17 lineages; 1.41 lineages/site; 12 sites; Figure 4). The difference is significant (χ^2^ = 11.43; *p* = .0007) even when excluding the site CH‐4 (Figures 2 and 4) characterized by an unusually high number of lineages (χ^2^ = 4.492; *p* = .0340). For instance, four sites from LGS of 140–150 days displayed five private lineages or more (two sites in SF and two sites in CH network), while all sites from LGS of 150–160 days harbored three lineages or less.

One site (CH‐4; Figures 2 and 4; Table 1) located at the northern limit of the LGS of 140–150 days clearly appears as an outlier as it displayed a very high diversity. It contained 15 hybrid lineages including 13 private lineages, identified from 24 hybrid individuals. However, 11 out of the 13 private lineages were represented by a single individual. This indicates that diversity of this population (in terms of the number of lineages) has been largely underestimated due to the presence of several lineages with low frequency. Random samplings from simulated populations revealed that the population should include at least 50 different lineages to produce a similar diversity (as a mean of 100 random samplings of 24 individuals). The exceptionally high diversity of CH‐4 site suggests very recent hybridization events compared to those occurring in the other sites of the survey.

**Figure 5:**
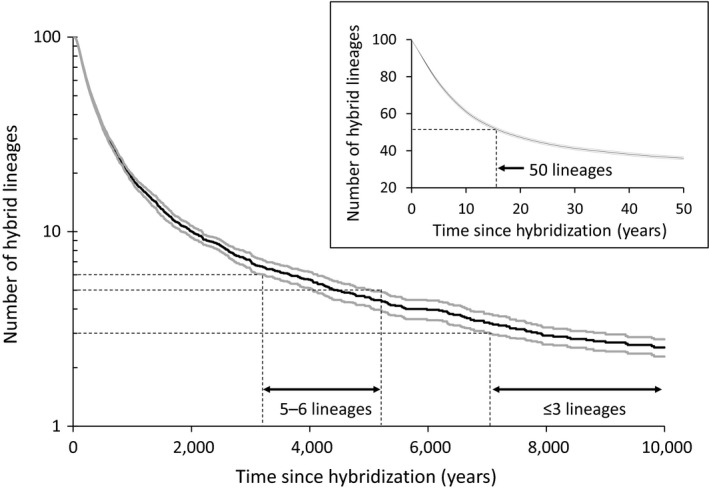
Reduction in the number of hybrid lineages through time by simulating random processes of lineage sorting. Number of lineages (mean and 95% confidence intervals) remaining in the whole system as a function of time since hybridization. Inferences of the time periods when hybridization may have occurred are indicated by “5–6 lineages” referring to diversity observed at some sites from LGS of 140–150 days and “<3 lineages” referring to the maximal diversity detected in sites from LGS of 150–160 days. Insert indicates the number of lineages remaining in each population as a function of time since hybridization. “50 lineages” refers to the expected diversity at CH‐4 site

Simulations of the random processes related to lineage sorting clearly illustrated the reduction in the number of lineages through time, when hybridization is a punctual event rather than a continuous process (Figure 5). Diversity remains high in the whole system after 100 generations (ca. 80 lineages). However, the number of lineages per population rapidly decreased to 50 lineages per population (the number expected in site CH‐4) in less than 20 generations.

A system comparable to LGS of 140–150 days with a diversity of 5–6 lineages at some sites is detected when hybridization occurred between ca. 3,000 and 5,000 years BP. A system comparable to LGS of 150–160 days (the southernmost sites) with a diversity of three lineages or less is detected when hybridization occurred >7,000 years BP. Finally, hybridization that occurred 10,000 years ago (the maximum of generations corresponding to postglacial expansion) still displays diversity and some systems could maintain up to three lineages.

## 4 DISCUSSION

The aim of this study was first to assess whether temperature (or associated environmental conditions) affected reproductive isolation between *C. eos* and *C. neogaeus* (spatial pattern) and second whether climate warming (previous or current episodes) resulted in a latitudinal progression of hybridization through time (temporal pattern). The clonal hybrids that keep track of hybridization between those species are therefore a useful biological indicator through which to infer the effects of climate warming.

Our 500 km latitudinal survey revealed the presence of 78 distinct *C. eos–neogaeus* hybrid lineages. This striking result contrasts with diversity from the other regions of North America surveyed so far. Previous studies reported a single or a very low number of widespread lineages per region (Angers & Schlosser, 2007; Doeringsfeld, Schlosser, Elder, & Evenson, 2004; Elder & Schlosser, 1995; Goddard et al., 1989; Vergilino et al., 2016). Such a low diversity in very large areas is typical of mtDNA diversity of species that colonized regions covered by the ice sheet during the Pleistocene (Bernatchez & Wilson, 1998). Applied to the context of clonal fishes, low diversity has been associated with postglacial expansion of a single (or a few) clones from glacial refuges (Angers & Schlosser, 2007).

Results of our study revealed that most of the hybrid lineages (70 lineages) were specific to a single site or were detected in a few geographically close sites that shared the same lineage assemblage. The very high diversity of lineages and their restricted distribution are consistent with the hypothesis that hybridization events occurred locally. Genetic analyses provided another support to this hypothesis as the multilocus genotype of hybrids from RI, YA, and SF networks matched that of *C. eos* sympatric populations, one of the species involved in hybridization (Vergilino et al., 2016).

The latitudinal pattern of diversity is an additional argument to reject the hypothesis that hybrids have colonized this region. The increase in hybrid lineage diversity with latitude in the southern half of the survey (diversity in LGS 140–150 > LGS 150–160, Figures 3 and 4) was unexpected in a postglacial colonization scenario. Indeed, biogeographic and phylogeographic studies reported a consistent pattern of rarefaction in species and haplotype diversity associated with barriers and founder events during northward postglacial expansion (Bernatchez & Wilson, 1998; Legendre & Legendre, 1984).

Altogether, these results support the rejection of the postglacial colonization and suggest that these 70 private lineages are the result from local hybridization. The next step is therefore to investigate local factors that affected the latitudinal variation in lineage diversity.

### 4.1 Spatial pattern of hybridization

The spatial distribution of private hybrid lineages is consistent with a temperature or environmental condition effect on hybridization. While *C. eos* and *C. neogaeus* are (or were) sympatric in most of the hydrographic networks analyzed, the diversity of private hybrid lineages strongly varied latitudinally. We observed a higher proportion of sites where hybridization occurred locally and a higher number of private lineages in the southern half of the survey than in the northern sites.

This suggests that sympatry alone is not sufficient to explain hybridization between *C. eos* and *C. neogaeus* species. For instance, both species were found in sympatry in eight sites (SJ‐4, SF‐9, OU‐1, OU‐2, OU‐3, LO‐2, RM‐3, and MI‐1; Table 1) that were sampled during two consecutive years while no private lineages were detected; those sites were located in the northernmost networks*.* Moreover, the number of private lineages is anecdotic (0.45/site) in the northern half of the survey, even in the presence of both species in sympatry. The low number of private lineages indicated that premating barriers have not been disturbed yet in regions characterized by a LGS shorter than 140 days.

### 4.2 Effect of temperature on reproductive barriers

Hybridization may result from the decline of one species. When the abundance of conspecific mates decreases, the reproduction with more abundant heterospecific males is then favored (Hubbs, 1955). This hypothesis finds support in the quasi‐absence of *C. neogaeus* from the networks of southeastern Quebec (RI, YA, and SF; Table 1), while this species had historically been present in those networks (Vergilino et al., 2016). Hybridization may have occurred during the demographic decline preceding the extinction of *C. neogaeus.* Moreover, the extirpation of this species from SF networks prevents further hybridization in these sites.

However, hybridization events were detected in sites in the CH hydrographic network, where *C. neogaeus* is still present. This suggests that hybridization is probably not only the result of the demographic decline of *C. neogaeus*, but also of the modification of other reproductive barriers by environmental conditions associated with climate warming. Several studies recently confirmed that climate warming strongly modifies the reproductive phenology of several species (e.g., Benard, 2015; Carey, 2009; Wegge & Rolstad, 2017). This may result in overlapping reproductive periods that increase the probability of hybridization. While change in the reproductive phenology of *C. eos* and *C. neogaeus* in response to climate warming remains to be experimentally demonstrated, such modification of the reproductive phenology has been observed in other fish species (Firkus, Rahel, Bergman, & Cherrington, 2018; Hovel, Carlson, & Quinn, 2017).

### 4.3 Temporal pattern of hybridization

We predicted that if local hybridizations were the consequence of climate warming, diversity of hybrids would vary latitudinally. Diversity of lineages from local hybridization events is expected to decrease as the time elapsed since the hybridization events increase, in the absence of additional hybridization. The increase in the number of lineages detected when moving northward along the southern half of the survey is consistent with this prediction.

The high hybrid diversity in the LGS of 140–150 days reflects the most recent historical hybridization events observed. This region also harbored four sites in which at least five distinct private lineages were detected, a diversity of lineages observed in no other sites or previous studies (Angers & Schlosser, 2007; Doeringsfeld et al., 2004; Elder & Schlosser, 1995; Goddard et al., 1989). Simulations indicated that a system maintaining such diversity originated from hybridization that should have occurred between ca. 3,000 and 5,000 years ago. This scenario is consistent with the Holocene Optimum time expected to occur ca. 6,000 to 3,550 years BP in southern Quebec (Comtois, 1982; Larochelle et al., 2018).

In spite of more exhaustive sampling (in terms of a larger longitudinal survey) performed in the LGS of 150–160 days (Leung et al., 2016; Vergilino et al., 2016), and a high number of lineages detected, the diversity of private lineages per site remains lower than in the northerly adjacent LGS category. This lower diversity suggests that hybridization took place earlier in the southernmost sites (RI, YA, and southern part of SF). Results of simulations indicated that hybridization in sites maintaining a maximum of three lineages likely occurred > 7,000 years BP. These events occurred long before (around 2,000 years) those observed in the sites of LGS of 140–150 days (northern part of SF and CH). While these simulations must be interpreted with caution, they provide a likely scenario illustrating the timing of hybridization events which occurred in southern Quebec during warming of the Holocene Climate Optimum period and the current warming episode.

### 4.4 Effect of current global warming

One site (CH‐4) revealed an exceptionally high number of lineages, one order of magnitude higher than in other sites. The unusually high diversity of CH‐4 contrasts with that of the other sites of the survey. The low diversity of those sites is expected to be representative of the population. For example, the same lineages were detected when sampling geographically close sites (Vergilino et al., 2016), indicating that most of the lineages were recovered. While 15 hybrid lineages including 13 private lineages were sampled in CH‐4, we estimated that this population should include at least 50 different lineages to produce diversity similar to our sampling. This suggests very recent (during the last decades) hybridization events occurred at CH‐4 site.

Effects of the current climate warming are suspected because of a significant increasing trend in mean water temperature and precipitation, associated with increasing temperature, has been observed over the past decades in southern Quebec (Hudon, Armellin, Gagnon, & Patoine, 2010; Yagouti, Boulet, Vincent, Vescovi, & Mekis, 2008; Zhang, Vincent, Hogg, & Niitsoo, 2000). As evidence of warming, the mean annual temperature recorded between 1960 and 2005 increased by ca. 1.0°C in the LGS of 140–150 and 150–160 days, while no significant changes were detected in the northern part of the sampling (Yagouti et al., 2008).

Results of the simulations revealed that 100 lineages produced by a local hybridization can rapidly decrease to 50 lineages per population (the number expected in site CH‐4) in less than 20 generations. This result suggests that a LGS of 140–150 days could serve as a reference for conditions triggering hybridization between the northern redbelly dace (*C. eos*) and the finescale dace (*C. neogaeus*).

In conclusion, the present study provides an empirical framework to assess how historical climate warming influenced hybridization between sympatric sister species. However, detection of contemporary hybridization close to the northern limit of historical hybridization suggests that the current warming episode is going beyond the limits of the Holocene Optimum, a conclusion recently reached by Porter et al. (2019) on warming in northwestern Canada. This suggests that organisms will soon have to face effects of the current climate warming they never experienced in the last era.

## CONFLICT OF INTEREST

None declared.

## Data Availability

All relevant data are within the paper.
